# Oxime Esters of 2,6-Diazaanthracene-9,10-dione and 4,5-Diazafluoren-9-one as Photo-induced DNA-Cleaving Agents

**DOI:** 10.3390/molecules17033370

**Published:** 2012-03-15

**Authors:** Shang-Shing P. Chou, Jui-Chi Juan, Shwu-Chen Tsay, Kuei Pin Huang, Jih Ru Hwu

**Affiliations:** 1 Department of Chemistry, Fu Jen Catholic University, New Taipei 24205, Taiwan; Email: a22022148@yahoo.com.tw; 2 Department of Chemistry, National Central University, Jhongli 32001, Taiwan; Email: tsay.susan@gmail.com (S-C.T.); koala790901@hotmail.com (K.P.H.); 3 Department of Chemistry, National Tsing Hua University, Hsinchu 30013, Taiwan

**Keywords:** photo-induced DNA-cleavage, oxime esters, 2,6-diazaanthracene-9,10-dione, 4,5-diazafluoren-9-one

## Abstract

Two series of oxime esters containing the 2,6-diazaanthracene-9,10-dione bis-(*O*-benzoyloxime) and 4,5-diazafluoren-9-one *O*-9-benzoyloxime moieties have been synthesized and tested as photo-induced DNA cleaving agents. All these compounds were found to cleave DNA upon irradiation with 312 nm UV light. The structure-activity relationship of these molecules for DNA cleavage was established. A plausible reaction mechanism is also proposed.

## 1. Introduction

Photocleavage of nucleic acids (DNA and RNA) can be very useful for molecular biological applications [1]. Organic molecules with DNA-cleaving ability are of great potential in the development of biotechnology and gene therapy [2]. A very important feature of this method is that all components can be mixed together without initiating the chemical reaction until it is irradiated [3]. If such “photocleavage agents” absorb light at wavelengths longer than 300 nm, nucleic acids will not be affected while selective excitation of the photocleavage agents can be achieved [4]. Since single-strand DNA damage is easily repaired by enzymatic processes [5], photocleavage of double-strand DNA molecules would be a more efficient tool for cancer therapy [6,7,8,9,10,11,12].

**Scheme 1:**
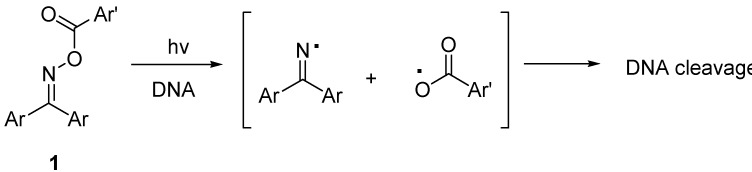
DNA cleavage by photolysis of oxime esters.

We were particularly interested in developing methods of DNA cleavage by radical species. A key feature is to facilitate the generation of radicals which are also reactive enough to cleave the DNA, as shown in the photolysis of *N*-aroyloxy-2-thiopyridones [13]. Along this line we have synthesized oxime esters **1** to cleave the calf thymus DNA upon UV irradiation (Scheme 1) [14]. 

The weak N–O bond of the oxime esters can be selectively cleaved to generate the iminyl and carboxy radicals which can then cause the cleavage of the DNA. The radicals were detected by EPR (electron paramagnetic resonance) spectroscopy. We incorporated the anthraquinone, fluoren-9-one, or thioxanthen-9-one moiety into the structure of the oxime esters as well as different substituents on the aromatic carboxyl group to determine their effects on the efficiency of DNA cleavage. Most of these compounds exhibited single-strand scission of DNA, but some anthraquinone derivatives caused double-strand scission. We also studied the photo-induced DNA cleavage by heteroaromatic oxime esters of anthraquinone [15], and discovered that one particular compound could cleave DNA at the concentration as low as 1.0 μM. Recently, anthracenone-based oxime esters have been reported to display strong antiproliferative activity against K562 leukemia cells [16]. *N*,*O*-Diacyl-4-benzoyl- *N*-phenylhydroxylamines, also having a weak N–O bond, were recently reported to produce single strand cleavage of DNA [17]. Photolysis of the carboxylic esters of *N*-acyl-*N*-phenylhydroxylamines generated the corresponding carboxylic acids by cleaving the N–O bond [18].

## 2. Results and Discussion

**Scheme 2:**
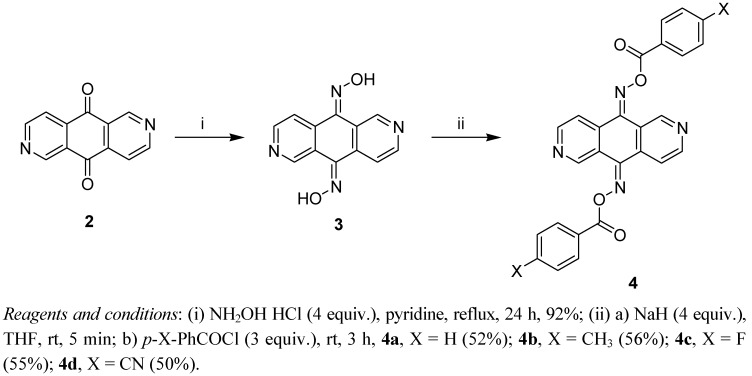
Synthesis of bis-oxime esters **4a**–**d**.

**Scheme 3:**
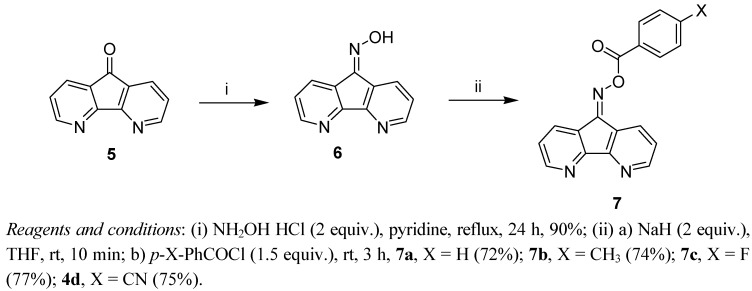
Synthesis of bis-oxmie esters **7a**–**d**.

The reaction of 2,6-diazaanthracene-9,10-dione (**2**) [19] with hydroxylamine hydrochloride (4 equiv.) in refluxing pyridine for 24 h gave the bis-oxime **3** in good yield. Treatment of compound **3** in anhydrous THF with sodium hydride (4 equiv.) at room temperature followed by the reaction with benzoyl chloride derivatives afforded the corresponding bis-oxime esters **4a**–**d** in fair yields (Scheme 2). 4,5-Diazafluoren-9-one (**5**) [20] was similarly converted to its oxime **6** and oxime esters **7a**–**d** (Scheme 3).

**Figure 1:**
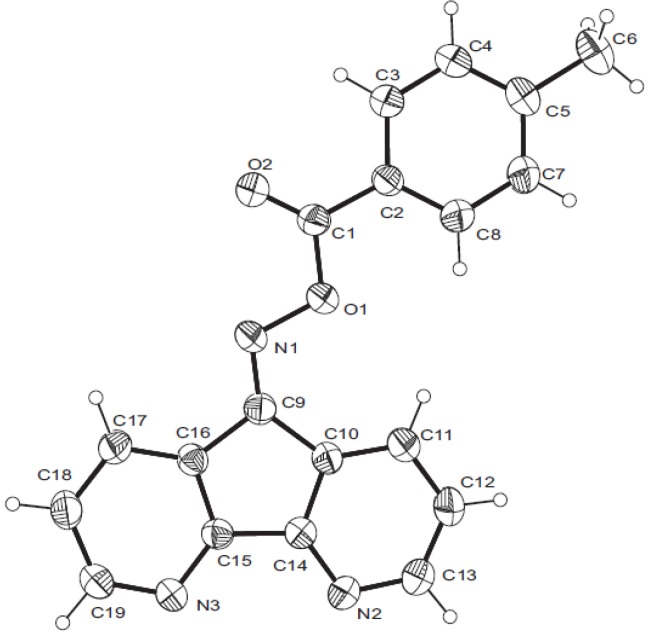
X-ray crystal structure of compound **7b**.

The characteristic ^1^H NMR absorptions for compounds **4a**-**d** and **7a**-**d** are provided in the Experimental section. The number of different carbons in the ^13^C-NMR spectra of compounds **4a**-**d** clearly demonstrates that they only have the *anti* configuration. The X-ray crystal structure of compound **7b** (Figure 1) shows the more stable *cisoid* conformation of the C=O and N-O group [21].

**Figure 2:**
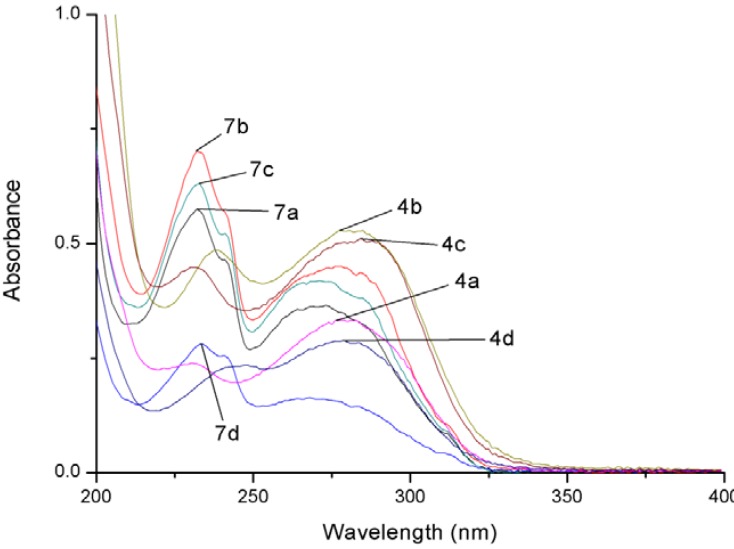
UV-VIS absorption spectra for compounds **4a**–**d** and **7a**–**d**.

The UV-VIS absorption spectra for compounds **4a**–**4d** and **7a**–**7d **were measured in CH_2_Cl_2_ (Figure 2), and their λ_max_ and molar absorptivity (ε) values are listed in Table 1. It can be seen that compounds **4a**–**d** and **7a**–**d **all have some absorption at the 312 nm normally used for the photo-induced DNA cleavage.

**Table 1:** λ_max_ and molar absorptivity (ε) for compounds **4a**–**d** and **7a**–**d**.

Compound	λ_max_ (ε)
**4a**	230 (23888), 280 (32944)
**4b**	238 (48714), 277 (52709)
**4c**	230 (44888), 283 (50593)
**4d**	248 (23504), 279 (28855)
**7a**	233 (57149), 273 (36500)
**7b**	232 (70174), 276 (45050)
**7c**	233 (63071), 271 (41946)
**7d**	234 (28102), 268 (16377)

**Figure 3:**
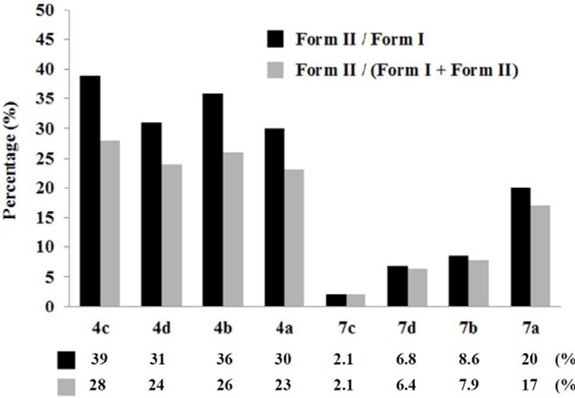
DNA cleaving abilities of compounds **4a**–**d** and **7a**–**d** (100 μM).

**Figure 4:**
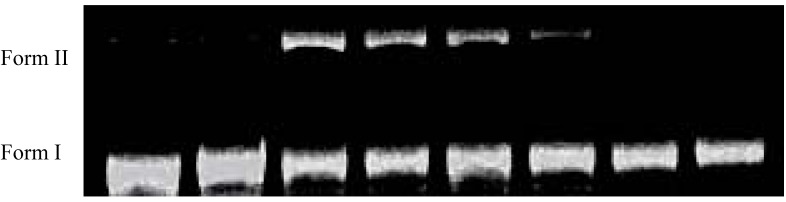
Dose measurement of compound **4c** for DNA cleavage. Lane 1, DNA at 312 nm for 2 h; Lane 2, DNA and **4c** (500 μM) in the dark; Lanes 3–8, DNA and 500, 250, 100, 50, 25, 12.5 μM of **4c**, individually, at 312 nm for 2 h.

The oxime esters **4a**–**d** and **7a**–**d** were individually irradiated with UV light (312 nm) at the concentration of 100 μM in phosphate buffers (pH 6.0) and 2.5% DMSO containing the supercoiled circular *ϕX174 *RFI DNA (form I; 50 μM/base pair) under aerobic conditions at room temperature for 2 h. The cleavage results from gel electrophoresis on 1% agarose with ethidium bromide staining for compounds **4a**–**d** and **7a**–**d** are shown in Figure 3. It can be seen that all these compounds nicked the supercoiled circular DNA to give the relaxed circular (*i.e.*, form II) DNA, and the cleaving ability of compounds **4a**–**d** was greater than of compounds **7a**–**d**. A simple explanation for the higher cleaving effect of compounds **4a**–**d** is that they have bis-oxime ester moieties, whereas compounds **7a**–**d** are mono-oxime esters. However, other factors such as degree of intercalation, polarity, and steric effect, *etc*. may also be involved. It is interesting to note that the substituent effect in these two series of compounds is almost reversed: for compounds **4a**–**d**, F > CH_3_ > CN > H; for compounds **7a**–**d**, H > CH_3_ > CN > F. Among these eight compounds, compound **4c** exhibited the best results, so we carried out further cleavage experiments with different concentrations of compound **4c** (Figure 4). In lane 1, without compound **4c**, DNA was not decomposed by irradiation with 312 nm of UV for 2 h. In lane 2, in the presence of 500 μM of compound **4c**, DNA cleavage did not occur in the dark. However, in lanes 3–6, in the presence of 500, 250, 100 or 50 μM of compound **4c**, significant amount of the relaxed circular (*i.e.*, form II) DNA was obtained. Thus the UV light functioned as a “trigger” to initiate the DNA scission process. However, with 25 or 12.5 μM of compound **4c**, DNA cleavage did not occur.

**Scheme 4:**
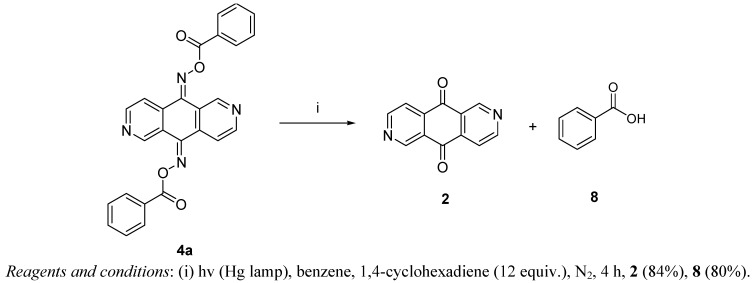
Photolysis of compound **4a** in the presence of 1,4-cyclohexadiene.

**Scheme 5:**
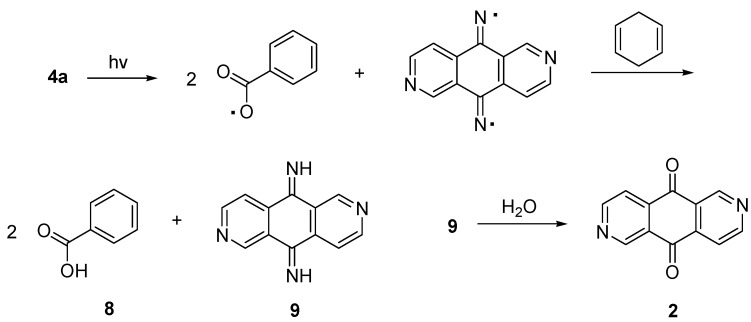
Proposed mechanism for the photolysis of compound **4**.

We have also carried out the photolysis of compound **4a** with a medium pressure mercury lamp (222–366 nm) in benzene under nitrogen using 1,4-cyclohexadiene [22] as the radical scavenger (Scheme 4). The products were purified by column chromatography to give 2,6-diazaanthracene-9,10-dione (**2**) and benzoic acid in good yields. These DNA-cleaving processes were further investigated in control experiments with oxime ester **4c** and **7a**, respectively, by addition of sodium azide (100 to 500 mM) as a scavenger of singlet oxygen [23,24]. The presence of singlet oxygen may contribute to the DNA damage [25,26].We found that the DNA cleavage results did not show obvious reduction after the addition of sodium azide. This outcome implies that singlet oxygen did not participate in these DNA-cleaving processes. In order to explain the photolysis products a plausible mechanism is proposed (Scheme 5) [27,28,29]. The weak N–O bond is first cleaved by the UV light to generate the bis-iminyl radical and benzoyloxy radical which can then abstract hydrogen atoms from 1,4-cyclohexadiene to give the bis-imine **9** and benzoic acid (**8**). The bis-imine **9** can be easily hydrolyzed to 2,6-diaza- anthracene-9,10-dione (**2**) during workup. Based on the photolysis results, we propose that the DNA cleavage is initiated by the homolytic cleavage of the weak N–O bond of oxime esters **4** or **7** to generate the bis-iminyl radical and benzoyloxy radical, which could then abstract hydrogen atom from the sugar moiety of the DNA. 

## 3. Experimental

### 3.1. General

Melting points were determined with a SMP3 melting apparatus, and were uncorrected. Infrared spectra were recorded with a Perkin-Elmer Spectrum 1600 FT-IR spectrometer. NMR spectra were recorded on a Bruker AV-300 spectrometer. Me_4_Si (δ 0.00 ppm) and the center of the CDCl_3_ triplet (δ 77.00 ppm) were used as the internal standard for ^1^H- and ^13^C-NMR spectra, respectively. All NMR chemical shifts are reported as δ values in parts per million (ppm) and coupling constants (*J*) are given in Hertz (Hz). High resolution mass spectra (HRMS) were measured with a JEOL JMS-SX102A mass spectrometer. UV-VIS spectra were taken with a Scino S-3100 spectrophotometer. Flash column chromatographic purifications were performed using Merck 60 H silica gel.

### 3.2. General Procedure for the Preparation of Oximes **3** and **6**

A mixture of compound **2** or **5** (0.48 mmol) and hydroxylamine hydrochloride (4 equiv. for **2**, 2 equiv. for **5**) in pyridine (5 mL) was heated under reflux for 24 h. After cooling to room temperature, water (50 mL) was added, and the solid formed was collected by vacuum filtration and was washed sequentially with CH_2_Cl_2_ (50 mL) and acetone (10 mL), and then dried under vacuum to give products **3** or **6**.

#### 3.2.1. 2,6-Diazaanthracene-9,10-dioxime (**3**)

Yield: 105 mg (92%); yellowish white solid: mp 236 °C (decomp); IR (film) *v* 3369, 3123, 2806, 2688, 2556, 1824, 1636, 1593, 1541, 1493, 1462, 1408, 1331, 1282, 1239, 1201, 1157, 1070, 1032, 949, 833, 796 cm^−1^; ^1^H-NMR (DMSO-d_6_) δ 9.90 (1H, s), 8.68 (2H, d, *J* = 5.1 Hz), 7.97 (2H, d, *J* = 4.5 Hz); ^13^C-NMR (DMSO-d_6_) δ 151.1, 150.0, 141.6, 139.2, 121.0, 118.0; EI-MS (relative intensity) *m/z *240 (M^+^, 1), 256 (2), 240 (1), 129 (2), 86 (4), 79 (32), 78 (100), 63 (99), 62(6), 61(17); Exact mass calcd for C_12_H_8_N_4_O_2_ *m/z* 240.0647 (M^+^), EI-HRMS *m/z *240.0641.

#### 3.2.2. 4,5-Diazafluoren-9-oxime (**6**)

Yield: 97 mg (90%); white solid: mp 253.2–254.4 °C; IR (film) *v* 3133, 3032, 2761, 2561, 1624, 1564, 1494, 1466, 1398, 1351, 1287, 1158, 1004, 948, 817, 751, 701, 638 cm^−1^; ^1^H-NMR (DMSO-d_6_) δ 8.72 (2H, dd, *J* = 9.0, 4.5 Hz), 8.65 (1H, dd, *J* = 7.8, 1.2 Hz), 8.17 (1H, dd, *J* = 7.5, 0.9 Hz), 7.54–7.45 (2H, m); ^13^C-NMR (DMSO-d_6_) δ 157.6, 156.7, 151.5, 151.0, 146.5, 135.5, 130.7, 128.6, 124.9, 124.3, 124.1; EI-MS (relative intensity) *m/z *197 (M^+^, 1), 197 (1), 80 (4), 79 (26), 78 (100), 63 (57), 61 (12), 45 (13), 31 (26), 17 (14); Exact mass calcd for C_11_H_7_N_3_O *m/z* 197.0589 (M^+^), EI-HRMS *m/z *197.0587.

### 3.3. General Procedure for the Prepartion of Oxime Esters **4** and **7**

To a mixture of compound **3** or **6** (0.20 mmol) in THF (3.5 mL) at room temperature was added NaH (60% in oil, 4 equiv. for **3**, 2 equiv. for **6**). After stirring for 5 min, the appropriate acid chloride (3 equiv for **3**, 1.5 equiv. for **6**) was added in one portion. The mixture was stirred for 3 h, and then the solvent was evaporated under vacuum. The residue was dissolved in CH_2_Cl_2_ (30 mL) and was washed with water (20 mL × 3), dried (MgSO_4_), and evaporated under vacuum. The crude products **4** or **7** were rinsed with hexane, and then recrystallized from CH_2_Cl_2_/hexane. 

#### 3.3.1. 2,6-Diazaanthracene-9,10-dione bis-(O-benzoyloxime) (**4a**)

Yield: 39 mg (52%); white solid: mp 214–215 °C; IR (film) *v* 3051, 1774, 1573, 1418, 1264, 1234, 1152, 1040, 1023, 990, 896, 732, 702 cm^−1^; ^1^H-NMR see Table 2; ^13^C-NMR (CDCl_3_) δ 163.3, 152.3, 151.6, 149.5, 139.6, 134.3, 130.2, 129.2, 127.7, 121.4, 120.1; EI-MS (relative intensity) *m/z *448 (M^+^, 1), 226 (5), 208 (5), 122 (8), 105 (100), 84 (6), 77 (37), 51 (5); Exact mass calcd for C_26_H_16_N_4_O_4_ *m/z* 448.1172 (M^+^), EI-HRMS *m/z *448.1167.

#### 3.3.2. 2,6-Diazaanthracene-9,10-dione bis-[O-(4-methylbenzoyl)oxime] (**4b**)

Yield: 44 mg (56%); white solid mp 224–225 °C; IR (film) *v* 3037, 1767, 1612, 1574, 1411, 1332, 1237, 1179, 1151, 1039, 983, 915, 866, 849, 800, 769, 737, 682 cm^−1^; ^1^H-NMR see Table 2; ^13^C-NMR (CDCl_3_) δ 163.3, 152.1, 151.5, 149.2, 145.3, 139.5, 130.2, 129.8, 124.8, 121.4, 120.0, 21.9; EI-MS (relative intensity) *m/z* 476 (M^+^, 1), 331 (11), 330 (8), 258 (40), 253 (15), 211 (13), 136 (13), 119 (100), 91 (39), 89 (21), 65 (11); Exact mass calcd for C_28_H_20_N_4_O_4_ *m/z* 476.1485 (M^+^), EI-HRMS *m/z *476.1477.

#### 3.3.3. 2,6-Diazaanthracene-9,10-dione bis-[O-(4-fluorobenzoyl)oxime] (**4c**)

Yield: 44 mg (55%); white solid: mp 217–218 °C (decomp); IR (film) *v* 3055, 2981, 1777, 1602, 1505, 1421, 1264, 1158, 1147, 1029, 991, 896, 828, 721, 703 cm^−1^; ^1^H-NMR see Table 2; ^13^C-NMR (CDCl_3_) δ 162.3, 152.4, 151.4, 149.6, 139.5, 132.9, 132.8, 124.0, 121.4, 120.1, 116.7, 116.5; EI-MS (relative intensity) *m/z *484 (M^+^, 1), 123 (9), 79 (46), 78 (100), 63 (89), 61 (16); Exact mass calcd for C_26_H_14_F_2_N_4_O_4_ *m/z* 484.0983 (M^+^), EI-HRMS *m/z *484.0981.

#### 3.3.4. 2,6-Diazaanthracene-9,10-dione bis-[O-(4-cyanobenzoyl)oxime] (**4d**)

Yield: 42 mg (50%); white solid: mp 209–210 °C; IR (film) *v* 3101, 3053, 2228, 1777, 1607, 1567, 1535, 1402, 1328, 1291, 1229, 1179, 1145, 1039, 978, 914, 853, 789, 749, 680 cm^−1^; ^1^H-NMR see Table 2; ^13^C-NMR (CDCl_3_) δ 161.8, 152.7, 151.3, 150.3, 139.3, 133.0, 131.6, 130.6, 121.2, 120.2, 117.9, 117.6; EI-MS (relative intensity) *m/z *498 (M^+^, 1), 331 (28), 330 (21), 259 (17), 258 (100), 208 (22), 182 (17), 147 (27), 130 (64), 102 (22); Exact mass calcd for C_28_H_14_N_6_O_4_ *m/z* 498.1077 (M^+^), EI-HRMS *m/z *498.1080.

**Table 2:** The characteristic ^1^H-NMR absorptions for compounds **4a**–**d** *^a^*. 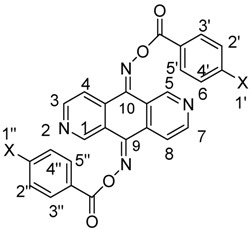

	**4a ** (X = H)	**4b ** (X = CH_3_)	**4c ** (X = F)	**4d ** (X = CN)
H-1, H-5	9.84 (s)	9.83 (s)	9.79 (s)	9.76 (s)
H-3, H-7	8.94 (d, 5.2)	8.92 (d, 5.1)	8.94 (d, 5.1)	8.97 (d, 5.2)
H-4, H-8	8.34 (d, 5.2)	8.32 (d, 5.1)	8.33 (dd, 5.1, 0.6)	8.32 (d, 5.2)
H-3′, H-5′, H-3′′, H-5′′	8.15 (dd, 7.2, 1.2)	8.04 (d, 8.1)	8.21–8.15 (m)	8.25 (d, 8.1)
H-1′, H-1′′	7.69 (br t, 7.3)	2.47 (s)	–	–
H-2′, H-4′, H-2′′, H-4′′	7.57 (br t, 7.3)	7.36 (d, 8.1)	7.29–7.21 (m)	7.88 (d, 8.1)

*^a^* The data are expressed as chemical shift in δ (splitting pattern, coupling constant in Hz).

#### 3.3.5. 4,5-Diazafluoren-9-one O-9-benzoyloxime (**7a**)

Yield: 44 mg (72%); white solid: mp 184–185 °C; IR (film) *v* 3055, 2981, 1754, 1596, 1563, 1449, 1398, 1264, 1239, 1169, 1079, 1045, 1021, 977, 891, 861, 819, 732, 703 cm^−1^; ^1^H-NMR see Table 3; ^13^C-NMR (CDCl_3_) δ 163.7, 160.3, 158.9, 154.5, 153.6, 153.4, 136.6, 134.1, 130.9, 130.1, 130.0, 129.1, 128.3, 125.3, 124.3, 124.2; EI-MS (relative intensity) *m/z *301 (M^+^, 31), 181 (37), 137 (32), 125 (44), 111 (72), 105 (85), 97 (100), 83 (80), 71 (84), 55 (96); Exact mass calcd for C_18_H_11_N_3_O_2_ *m/z* 301.0851 (M^+^), EI-HRMS *m/z *301.0850.

#### 3.3.6. 4,5-Diazafluoren-9-one O-9-(4-methylbenzoyl)oxime (**7b**)

Yield: 48 mg (74%); white solid: mp 232–233 °C; IR (film) *v* 3078, 3057, 2916, 1748, 1631, 1608, 1583, 1563, 1473, 1410, 1397, 1288, 1249, 1171, 1154, 1109, 1093, 1045, 1015, 977, 893, 861, 821, 748, 737, 684 cm^−1^; ^1^H-NMR see Table 3; ^13^C-NMR (CDCl_3_) δ 163.7, 160.2, 158.8, 154.1, 153.5, 153.2, 145.0, 136.5, 130.8, 129.9 (×2), 129.8, 125.3 (×2), 124.2 (×2), 21.8; EI-MS (relative intensity) *m/z *315 (M^+^, 15), 181 (32), 180 (13), 166 (17), 131 (10), 119 (100), 91 (30), 69 (17), 65 (12), 28 (13); Exact mass calcd for C_19_H_13_N_3_O_2_ *m/z* 315.1008 (M^+^), EI-HRMS *m/z *315.1008.

#### 3.3.7. 4,5-Diazafluoren-9-one O-9-(4-fluorobenzoyl)oxime (**7c**)

Yield: 50 mg (77%); white solid: mp 228–229 °C; IR (film) *v* 3069, 3013, 1764, 1603, 1562, 1396, 1281, 1243, 1156, 1114, 1098, 1041, 1011, 976, 945, 899, 852, 814, 792, 747, 678 cm^−1^; ^1^H-NMR see Table 3; ^13^C-NMR (CDCl_3_) δ 168.1, 164.7, 162.8, 160.4, 158.9, 154.6, 153.6, 136.5, 132.7, 132.6, 131.0, 130.0, 125.3, 124.6, 124.2, 116.5; EI-MS (relative intensity) *m/z *319 (M^+^, 15), 181 (27), 180 (10), 140 (14), 123 (100), 95 (22); Exact mass calcd for C_19_H_13_N_3_O_2_ *m/z* 319.0757 (M^+^), EI-HRMS *m/z *319.0754.

#### 3.3.8. 4,5-Diazafluoren-9-one O-9-(4-cyanobenzoyl)oxime (**7d**)

Yield: 50 mg (75%); white solid: mp 238–239 °C; IR (film) *v* 3056, 2959, 2926, 2851, 2231, 1767, 1731, 1593, 1563, 1471, 1394, 1236, 1175, 1097, 1054, 1016, 977, 892, 866, 814, 749, 705, 682 cm^−1^; ^1^H-NMR see Table 3; ^13^C-NMR (CDCl_3_) δ 162.3, 160.6, 159.0, 155.3, 154.0, 153.7, 136.5, 133.0, 132.3, 131.1 (×2), 130.4, 130.0, 125.2, 124.4, 124.2, 117.6; EI-MS (relative intensity) *m/z *326 (M^+^, 24), 181 (90), 180 (31), 147 (26), 130 (100), 123 (26), 102 (32), 57 (23); Exact mass calcd for C_19_H_10_N_4_O_2_ *m/z* 326.0804 (M^+^), EI-HRMS *m/z *326.0799.

**Table 3:** The characteristic ^1^H-NMR absorptions for compounds **7a**–**d***^a^*. 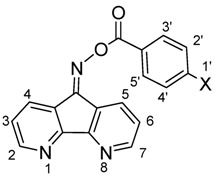

	**7a ** (X = H)	**7b ** (X = CH_3_)	**7c ** (X = F)	**7d** (X = CN)
H-2, H-7	8.80 (dd, 4.8, 0.9)	8.83–8.78 (m)	8.83–8.78 (m)	8.85–8.81 (m)
H-5	8.56 (dd, 7.8, 1.2)	8.58 (dd, 7.6, 1.3)	8.52 (dd, 7.6, 1.0)	8.47 (dd, 6.6, 1.0)
H-4	8.35 (dd, 7.5, 0.9)	8.36 (dd, 7.8, 1.2)	8.35 (dd, 7.5, 0.9)	8.36 (dd, 6.3, 1.0)
H-3′, H-5′	8.19 (d, 7.5)	8.09 (d, 8.1)	8.22 (dd, 8.7, 5.4)	8.31 (d, 8.4)
H-1′	7.72 (dd, 7.5, 7.5)	2.50 (s)	–	–
H-2′, H-4′	7.60 (dd, 7.8, 7.2)	8.09 (d, 8.1)	7.44–7.36 (m)	7.92 (d, 8.4),
H-3, H-6	7.43–7.35 (m)	7.43–7.36 (m)	7.29 (t, 8.4)	7.44–7.38 (m)

*^a^* The data are expressed as chemical shifts in δ (splitting pattern, coupling constant in Hz).

### 3.4. General Procedures for DNA-Cleavage by Use of Oxime Esters

A reaction mixture (10.0 μL), containing supercoiled circular *ϕX174* RFI DNA stock solution (form I, 50.0 μM/base pair) and an oxime ester (100 μM), was dissociated in the phosphate buffers (pH 6.0) and DMSO (0.25 μL) in a Pyrex vial. It was then preincubated at 37.0 °C for 30.0 min and irradiated with UV light (312 nm, 1.43 mW/cm^2^) under aerobic conditions at room temperature for 2.0 h. After addition of gel-loading buffer (2.50 μL containing 0.25% bromophenol blue, 0.25% xylene cyanol, and 30.0% glycerol), the reaction mixture was loaded on a 1.0% agarose gel with ethidium bromide staining. The electrophoresis tank was attached to a power supply at a constant current (~100 mA). The gel was visualized by 312 nm UV transilluminator and photographed by a Canon PowerShot S5 IS digital camera. Quantitation of DNA-cleavage was performed by integration of the optical density as a function of the band area by use of a Scion image beta 4.03 program.

### 3.5. Photolysis of 2,6-Diazaanthracene-9,10-dione bis-(O-benzoyloxime) (**4a**)

A mixture of compound **4a** (40 mg, 0.089 mmol) and 1,4-cyclohexadene (0.10 mL, 1.057 mmol) in benzene (5 mL) was placed in a quartz tube and was irradiated with a medium pressure mercury lamp (222–366 nm) under nitrogen for 4 h. The solvent was then removed by a rotary evaporator, and the residue was dissolved in CH_2_Cl_2_ (10 mL) and was then treated with 5% NaOH (10 mL). The organic solution was concentrated and then purified by flash column chromatography using ethyl acetate/hexane (1:1) as eluent to give 2,6-diazaanthracene-9,10-dione (**2**, 15.8 mg, 84%). The aqueous solution was neutralized with 5% HCl, extracted with CH_2_Cl_2_ (10 mL), dried (MgSO_4_) and evaporated to give benzoic acid (**8**, 17.4 mg, 80%).

## 4. Conclusions

In conclusion, oxime esters **4a**–**d** and **7a**–**d** newly synthesized from 2,6-diazaanthracene-9,10-dione (**2**) and 4,5-diazafluoren-9-one (**5**) were found to possess DNA cleaving ability upon UV irradiation. All of these bis-oxime esters **4a**–**d** showed greater cleaving ability than the mono-oxime esters **7a**–**d**. Upon UV irradiation, the most potent compound **4c** caused significant amounts of single-strand cleavage at the concentration of 100 μM. Results from our mechanistic study indicate that the iminyl and carboxyl radical species are responsible for DNA nicking.
